# Genome-wide investigation of the AP2/ERF gene family in ginger: evolution and expression profiling during development and abiotic stresses

**DOI:** 10.1186/s12870-021-03329-3

**Published:** 2021-11-25

**Authors:** Haitao Xing, Yusong Jiang, Yong Zou, Xiaoling Long, Xiaoli Wu, Yun Ren, Yuan Li, Hong-Lei Li

**Affiliations:** 1 College of Landscape Architecture and life Science/Institute of special Plants, Chongqing University of Arts and Sciences, Chongqing, 402168 China; 2 Chongqing Key Laboratory of Economic Plant Biotechnology, Chongqing University of Arts and Sciences, Chongqing, 402168 China

**Keywords:** Ginger, ZoAP2/ERF, Inflorescence/rhizome development, Abiotic stress, Expression patterns

## Abstract

**Background:**

AP2/ERF transcription factors (TFs) constitute one of the largest TF families in plants, which play crucial roles in plant metabolism, growth, and development as well as biotic and abiotic stresses responses. Although the AP2/ERF family has been thoroughly identified in many plant species and several AP2/ERF TFs have been functionally characterized, little is known about this family in ginger (*Zingiber officinale* Roscoe), an important affinal drug and diet vegetable. Recent completion of the ginger genome sequencing provides an opportunity to investigate the expression profiles of AP2/ERF genes in ginger on a genome-wide basis.

**Results:**

A total of 163 AP2/ERF *g*enes were obtained in the *Z.officinale* genome and renamed according to the chromosomal distribution of the ZoAP2/ERF genes. Phylogenetic analysis divided them into three subfamilies, of which 35 belonged to the AP2 subfamily, 120 to ERF, three to RAV, and five to Sololist, respectively, which is in accordance with the number of conserved domains and gene structure analysis. A total of 10 motifs were detected in ZoAP2/ERF genes, and some of the unique motifs were found to be important for the function of ZoAP2/ERF genes. The chromosomal localization, gene structure, and conserved protein motif analyses, as well as the characterization of gene duplication events provided deep insight into the evolutionary features of these ZoAP2/ERF genes. The expression profiles derived from the RNA-seq data and quantitative reserve transcription (qRT-PCR) analysis of ZoAP2/ERFs during development and responses to abiotic stresses were investigated in ginger.

**Conclusion:**

A comprehensive analysis of the AP2/ERF gene expression patterns in various tissues by RNA-seq and qRT-PCR showed that they played an important role in the growth and development of ginger, and genes that might regulate rhizome and flower development were preliminary identified. In additionally, the ZoAP2/ERF family genes that responded to abiotic stresses were also identified. This study is the first time to identify the ZoAP2/ERF family, which contributes to research on evolutionary characteristics and better understanding the molecular basis for development and abiotic stress response, as well as further functional characterization of ZoAP2/ERF genes with an aim of ginger crop improvement.

**Supplementary Information:**

The online version contains supplementary material available at 10.1186/s12870-021-03329-3.

## Background

The APETALA 2/Ethylene-Responsive element binding Factor (AP2/ERF) family composes one of the largest plant-specific transcription factors (TFs) and plays essential roles in various biological processes [1, 2]. Its members are defined by the AP2/ERF domain, which comprises one or two AP2 DNA-binding domains with 60 to 70 conserved amino acid residues [3]. According to previous reports, two classification systems were used in the investigation and comparative analyses of the AP2/ERF family. Sakuma et al., (2002) divided the AP2/ERF family into five subfamilies: AP2, ERF, DREB, RAV and others in Arabidopsis. Furthermore, the ERF subfamily and DREB subfamily were further divided into six subgroups consisting of clades A1-A6 and clades B1-B6 in Arabidopsis [4]. Based on the exon/intron and motif structural analyses of each genes in the AP2/ERF family, Nakano et al., (2006) classified the AP2/ERF family into three major subfamilies: subfamily ERF with one conserved AP2 domain, subfamily AP2 with two AP2 domains, and subfamily RAV with a single AP2 domain and an additional DNA-binding domain B3 that exists in other plant-specific TFs and soloists [5]. Since the first AP2 gene (*AP2-1*) was found to determines the identity of perianth organs in flowers of Arabidopsis [6], a large number of AP2 genes have been identified in various plants. The AP2 subfamily plays significant roles in the regulation of plant growth and development, such as floral organ identity and development [7–9], leaf shape [10], and seed growth [11]. Ethylene response factors (ERFs) function downstream of the ethylene signaling pathway and have been proven to be involved in many processes, such as metabolic regulation [12–14], responses to biotic and environmental stresses [15–18] and plant development and growth [18, 19]. ERFs have also been involved in different hormone signal transduction pathways including cytokinin, ethylene, and abscisic acid [20], However, RAV TFs mainly participate in the regulation of leaf senescence and biotic and abiotic stress responses [21, 22].

Ginger (*Zingiber officinale* Roscoe) is an important medicinal material containing abundant gingerols and exhibiting many biological properties, including antioxidant, antimicrobial, and anti-inflammatory properties, which have various effects on the central nervous system [23, 24]. Jiang et al. (2005) have reported that the rhizome of ginger had the greatest concentrations of gingerols compared with other organs [25]. The concentration of gingerols increases along with rhizome development. However, the mechanisms of ginger rhizome initiation and expansion, as well as gingerols synthesis and accumulation, are still delusively mysterious. In addition, ginger seldom blossoms in natural cultivation and propagates by rhizome block as the “seed”. As the cultivation year increases, the “seed” vigor decreases and pathogenicity increases, which could cause great losses in reproduction. The environmental stresses significantly affect the growth and development of plant. Ginger can be injured by sunburn under high temperature, whereas low temperature can result in restrained growth. Ginger can survive under a certain extent of drought conditions; nevertheless, long-term droughts significantly cause growth inhibition and yield loss of rhizome. Additionally, ginger plant is usually severely damaged by salt. Exploring the mechanism of development and abiotic tolerance is necessity for novel cultivar in breeding.

At present, investigations and analyses of the AP2/ERF gene family have been performed in many plants at the whole genome scale, including Arabidopsis [5], rice [5, 26], popular [27], grape [28], peach [29], Chinese cabbage [30], apple [31], sesame [32], pear [33], pepper [34] and tartary buckwheat [35]. However, no information about the AP2/ERF family in ginger (*Z. officinale*) has been reported. Because of the importance of the AP2/ERF family genes in various physiological processes, it is of significance to systematically study the AP2/ERF family in ginger. The evolutionary characteristics and tissue-specific expression of the AP2/ERF gene family in ginger could be characterized taking the advantage of recently sequenced ginger genome [36]. In this study, we performed a comprehensive analyses of the AP2/ERF family in ginger, including gene structure, motif composition, chromosomal localization, and phylogenetic tree; elucidated the evolutionary relationships of ginger by comparing with *Arabidopsis thaliana*, *Solanum tuberosum*, *Oryza sativa* and *Musa acuminata* and investigated the expression profiles of AP2/ERF genes in various ginger tissues, particularly in the stage of rhizome expansion and floral organ formation. This study provides valuable clues for future investigations aiming at the functional characterization of the AP2/ERF family genes and can be utilized in the genetic improvement of ginger.

## Results

### Genome-wide identification of the AP2/ERF family in ginger

A total of 163 AP2/ERF family candidate genes were obtained using the hidden Markov model (HMM) method with the the AP2/ERF family (PF00847) as the query. The annotation of these genes was further checked using available ginger transcriptome data. Fifteen erroneously predicted AP2/ERF gene models were manually curated. According to the classification system reported by Nakano et al. (2006), we divided the AP2/ERF family into four subfamilies, AP2, ERF, RAV, and Soloist, in ginger. A total of 157 *ZoAP2/ERF* genes could be mapped on the chromosomes and were renamed from *ZoAP2#01* to *ZoAP2#35*, *ZoERF#001* to *ZoERF#114*, *ZoRAV*#*1* to *ZoRAV*#*3*, and *ZoAP2Solo#1* to *ZoAP2Solo# 5* based on chromosomal locations (Additional file 1: Fig S1). Six *ZoERF* genes (*Maker00067748, Maker00058996, Maker00044912, Maker00021402, Maker00004369* and *Maker00027389*) that could not be conclusively mapped to any linkage group were renamed *ZoERF#115*-*ZoERF#119*, respectively. The validated *ZoAP2/ERF* gene sequences are available in Additional file 2: Table S1.

Gene characteristics, including the coding sequence (CDS) length, protein molecular weight (MW), isoelectric point (pI), and conserved domain, were analyzed. Among the 163 ZoAP2/ERF proteins, ZoERF#003 and ZoERF#023 were identified to be the smallest proteins with 117 amino acids (aa), whereas the largest one was ZoAP2#02 (671 aa). The MW of the proteins ranged from 12.92 (ZoERF#23) to 73.07 kDa (ZoERF#02), and the pIs ranged from 4.58 (ZoERF#106) to 11.17 (ZoERF#005) (Additional file 2: Table S1).

### Multiple sequence alignment, phylogenetic analysis, and classification of ZoAP2/ERF genes

Multiple sequence alignment of ZoAP2/ERF proteins was established based on the AP2 domain involving approximately 60-70 aa and the B3 domain consisting of 100-120 aa. The sequence alignment of all AP2/ERF proteins showed that the YRGVR (7th to 11th aa), LG (52nd amino acid and 53rd aa), AA (62nd and 63rd aa) and YD (65th and 66th aa) elements were highly conserved. The WLG element (51st to 53rd aa) was more conserved in the ERF and RAV subfamilies than in the AP2 subfamily. In the AP2 subfamily, WLG elements (51st to 53rd aa) were converted into YLG elements(51st to 53rd aa)(Additional file 3: Fig. S2). These conserved amino acid profiles may contribute to the classification of AP2/ERF genes in other species.

**Fig. 1:**
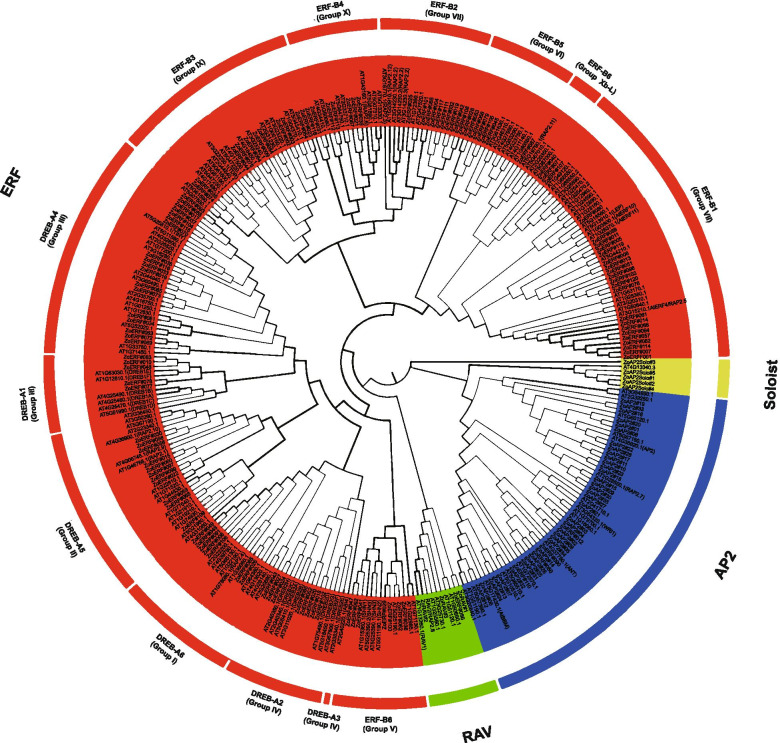
Unrooted phylogenetic tree representing the relationships among 163 AP2/ERF protein of ginger and Arabidopsis. The different colored arcs indicate different groups of the AP2/ERF family. AP2/ERF proteins from ginger with the prefix “Zo” indicate “*Zingiber officinale*”

To explore the phylogenetic relationship of AP2/ERF proteins in ginger, we constructed a phylogenetic tree using the maximum likelihood (ML) method based on the multiple sequence alignment of 166 *A. thaliana* AP2/ERF and 163 ginger AP2/ERF proteins. The phylogeny showed that AP2/ERF genes were grouped into four major categories, AP2, ERF, RAV, and Soloists, in ginger. Among the 163 candidate ZoAP2/ERF genes, 35 containing two AP2 domains were assigned to the AP2 subfamily; 120 containing a single AP2 domain were grouped in the lineage of the ERF subfamily; three encoding a single AP2 domain and a B3 domain were assigned to the RAV subfamily; and the other five Soloists. Interestingly, *ZoERF#110*, *ZoERF#050*, and *ZoERF#012* were also found to encode one AP2 domains, but they were distinct from the ERF subfamily and clustered with the AP2 subfamily (Fig. 1).

### Gene structure and motif composition of the ZoAP2/ERF family

**Fig. 2:**
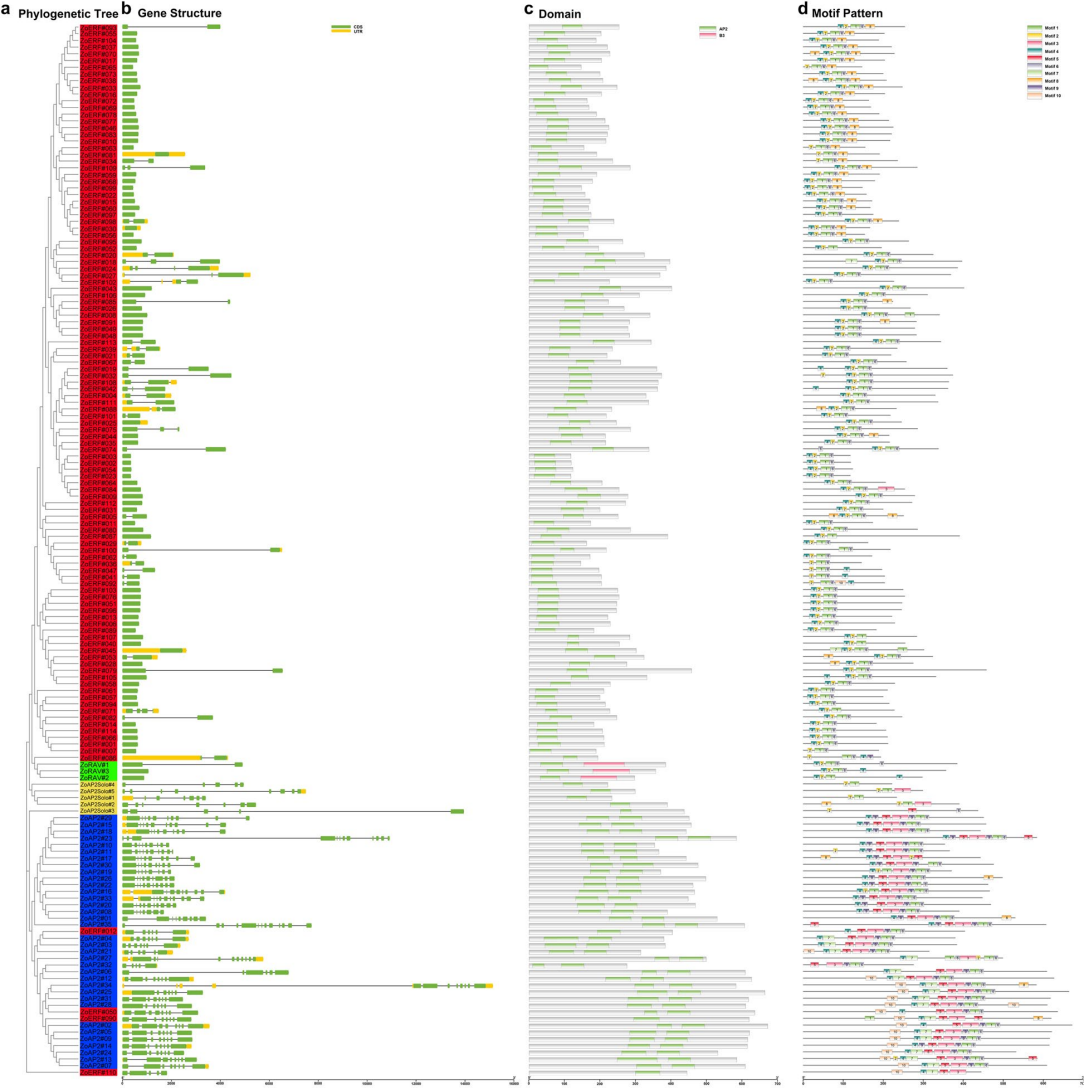
Phylogenetic relationships, gene structure and architecture of conserved protein motifs in AP2/ERF genes from ginger. **a** Phylogenetic tree based on the full-length sequences of ginger AP2/ERF proteins using MEGA X software. **b** Exon-intron structure of ginger AP2/ERF genes. Yellow boxes indicate 5′- and 3′- untranslated regions; green boxes indicate exons;and black lines indicate introns. **c** Motif composition of ginger AP2/ERF proteins. The motifs, numbers 1-10, are displayed in different colored boxes. The sequence information for each motif is provided in Additional file 3: Fig. S2. The protein length can be estimated using the scale at the bottom. **d** The AP2 domains are highlighted by green boxes and the B3 domain by pink boxes

By comparing the genomic DNA sequences, we obtained the intron and exon structure of ZoAP2/ERF genes (Fig. 2). The CDSs of all ginger AP2 subfamily genes were disrupted by introns, with exon numbers ranging from 5 to 13 (Fig. 2, Additional file 2: Table S1). Except the *ZoAP2#32* gene having 5 exons, the other members of the AP2 subfamily contained more than 6 exons. Overall, the number of exons was conserved in the AP2 subfamily, although the exon positions varied. Most members of the ERF subfamily and RAV subfamily contained only one exon with the AP2 domain located in the exonic region (Fig. 2b). In general, members with close relationships from the same subfamily shared similar exon numbers and exon lengths. Further analysis showed that ZoAP2/ERF proteins contained, at most, two characteristic regions (Fig. 2c). All ZoAP2/ERF proteins had a highly conserved AP2 domain in the N-terminals. This region, corresponding to the DNA binding region, was about 60-70 aa. The RAV subfamily contained a B3 domain composed of 100-120 aa. In general,,many conserved motifs were detected in TF protein sequences, which may be involved in activating the expression of genes as potential DNA binding sites. The motifs of the 163 ZoAP2/ERF genes were analyzed using the online MEME software to further study the characteristics of ZoAP2/ERF proteins (Additional file 4: Fig. S3). A total of 10 conserved motifs were found in the ZoAP2/ERF proteins (Fig. 2d). Motif-1, Motif-6 were found in the AP2 domain regions. Motif-1, Motif-3, Motif-4, Motif-6 and Motif-9 were detected in almost all AP2/ERF proteins. All the ERF subfamily genes contained Motif-1, Motif-2, Motif-4, and Motif-6.Motif-8 was detected in 29 ZoERF genes, Motif-9 was only present in two genes (*ZoERF#086* and *ZoERF#050*), Motif-10 in three genes (*ZoERF#092*, *ZoERF#90* and *ZoERF#110*), Motif-7 in four ZoERF genes (*ZoERF#045*, *ZoERF#110*, *ZoERF#090* and *ZoERF#018*), and Motif-3 only in *ZoERF#009*. In the AP2 subfamily, 17 genes contained six motifs including Motif-1, Motif-3, Motif-4, Motif-5, Motif-6, and Motif-9. Eleven ZoAP2 genes with Motif-10 and 15 ZoAP2 genes with Motif-7 were detected, respectively.

### Analysis of *cis*-acting elements of ZoAP2/ERF promoters

**Fig. 3:**
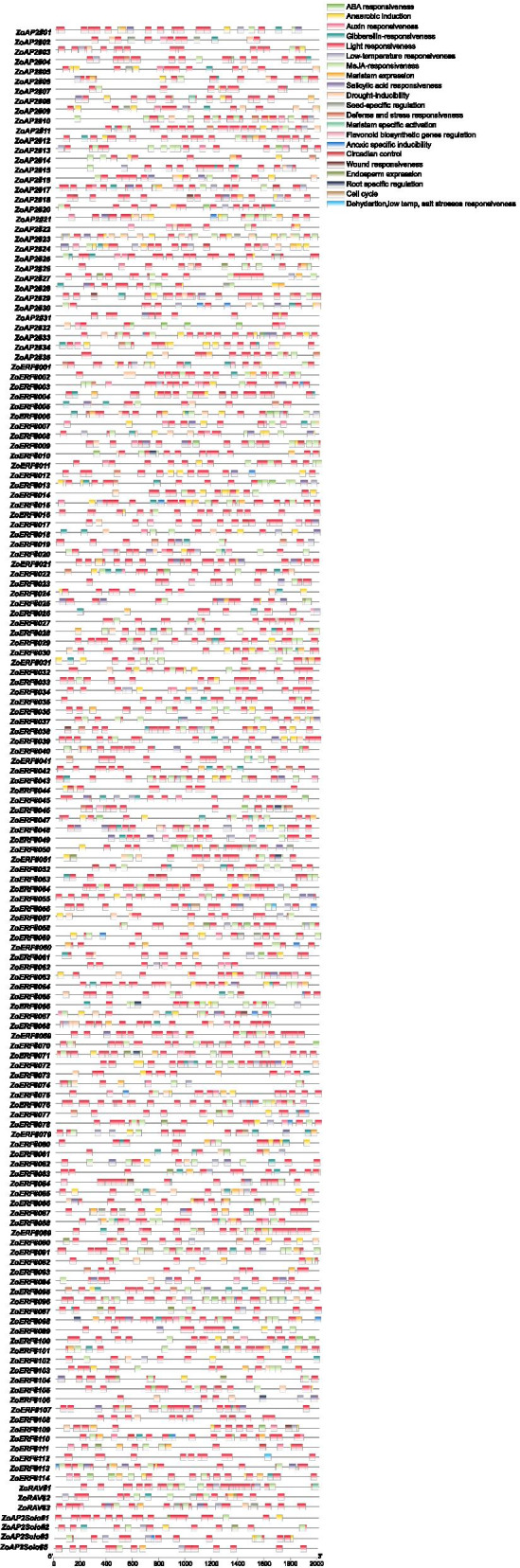
*Cis*-acting elements of ZoAP2/ERF promoters. Different colors represent different types of *cis*-acting elements

To further study the regulatory mechanism of the ZoAP2/ERF family genes during the abiotic stress responses, the upstream 2-kb sequences of 157 *ZoAP2/ERF* genes with complete domains were extracted from the ginger genome for *cis*-acting element analysis (Fig. 3 and Additional file 5: Table S2). Approximately 62 *cis*-acting elements that can be effectively expressed, and 223 elements that have clear functions were analyzed and revealed. Hormone response elements, included ABRE (abscisic acid responsiveness); GARE-motif, P-box, and TATC-box (gibberellin responsiveness); TCA-element and SARE (salicylic acid responsiveness); CGTCA-motif and TGACG-motif (MeJA-responsiveness); and TGA-element, TGA-box, AuxRR-core, AuxRE and TGA-element (auxin responsiveness). Abiotic stress response elements contained MBS (drought-inducibility), LTR(low-temperature responsiveness), WUN-motif(wound-responsive element), TC-rich repeats(defense and stress responsiveness), DRE (dehydration, low temperature, salt stress responsiveness), and GC-motif (anoxic specific inducibility). Light-responsive elements, included TCCC-motif, GATA-motif, chs-CMA1a, G-box, MRE, Box 4, Gap-box, TCT-motif, GA-motif, AE-box, LAMP-element, Box II, ATCT-motif, chs-CMA2a, I-box, AT1-motif, ATC-motif, ACE, chs-Unit 1 m1, ACA-motif, chs-CMA2b, L-box, CAG-motif, GTGGC-motif, GT1-motif, 3-AF1 binding site, Sp1, AAAC-motif, GATT-motif, TGGCA, sbp-CMA1c, 4 cl-CMA2b, Pc-CMA2a, and LS7. Other response elements, included GCN4-motif (endosperm expression), ARE (anaerobic induction), circadian (circadian control), AT-rich sequence (maximal elicitor-mediated activation), MSA-like (cell cycle), MBSI (flavonoid biosynthetic genes regulation), CAT-box and NON-box (meristem expression), motif I (root specific regulation), and RY-element (seed-specific regulation).The *cis*-regulatory elements, TC-rich repeats, TGA-element, ARE, ABRE, G-box, Box 4, LTR, CGTCA-motif and CGTCA-motif, were more abundant in most ZoAP2/ERF genes. WUN-motif that is related to wound responsiveness was uniquely found in *ZoAP2#29*, *ZoERF#086*, *ZoERF#069*, *ZoERF#038*, *ZoERF#109 and ZoAP2#18*. Seed-specific regulation *cis*-element, RY-element, was only found in *ZoERF#087*, *ZoERF#059*, *ZoAP2#23*, *ZoERF#050*, *ZoRAV#2*, *ZoAP2#02*, *ZoAP2#25*, *ZoAP2#05*, *ZoAP2#09*, *ZoAP2#12*, *ZoAP2#28*, *ZoRAV#3* and *ZoERF#070*. Motif I, a *cis*-element of root specific regulation, was found in *ZoERF#071*, *ZoERF#051*, *ZoERF#066*, *ZoERF#098*, *ZoERF#010*, *ZoERF#046* and *ZoERF#107*. MBSI, a *cis*-element of flavonoid biosynthetic gene regulation, was found in *ZoERF#100*, *ZoERF#077*, *ZoERF#026*, *ZoAP2#11*, and *ZoERF#046.* MSA like element that is related to cell cycle was unique to *ZoERF#079*, *ZoERF#050*, *ZoERF#091*, *ZoERF#046*, *ZoERF#107* and *ZoERF#056*.

### GO annotation of ZoAP2/ERF protein sequences

**Fig. 4:**
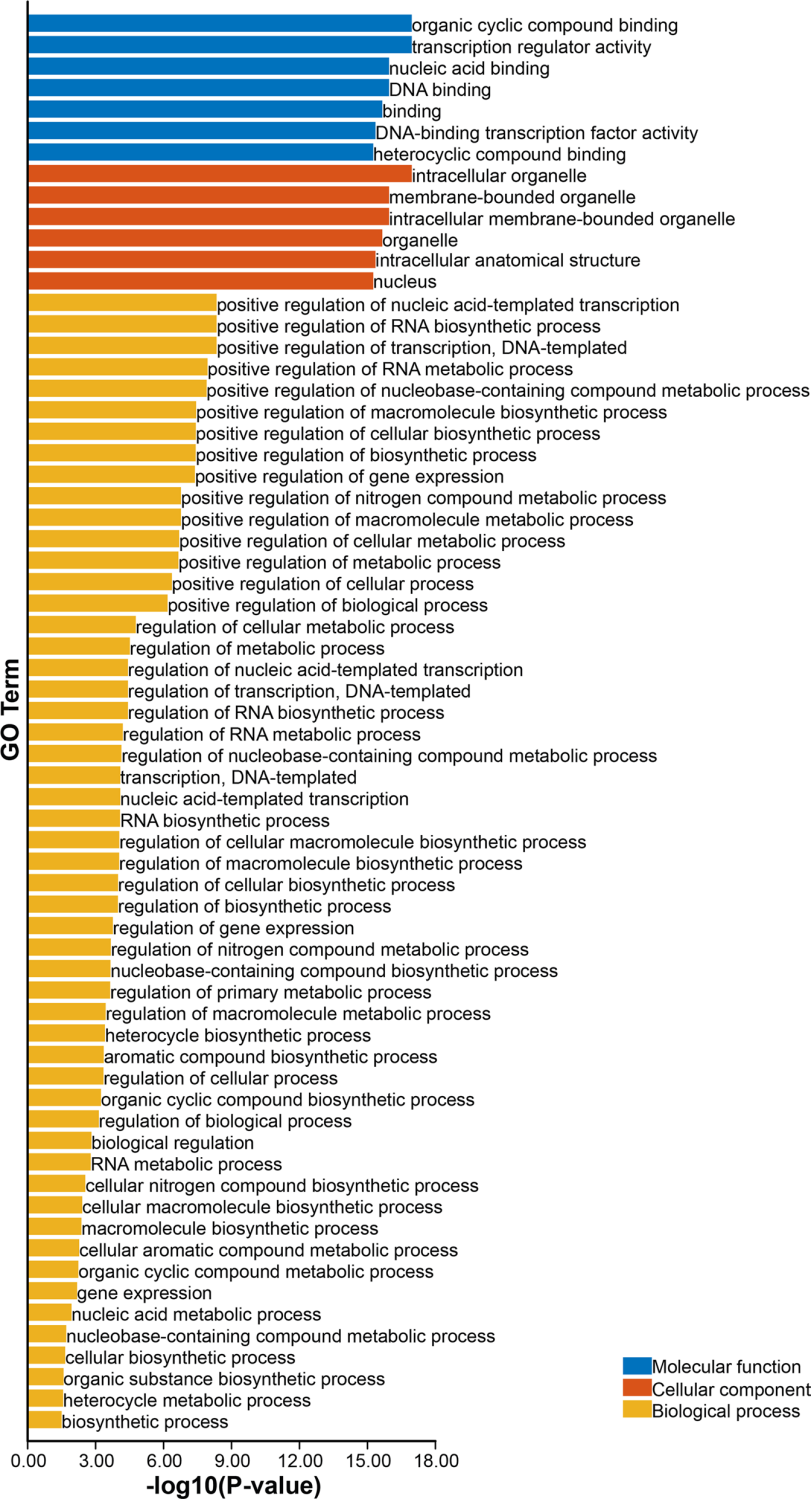
GO annotation of ZoAP2/ERF protein sequences

The gene ontology (GO) analysis was performed using rice protein sequences as the reference. The result showed the putative participation of ZoAP2/ERF proteins in diverse biological, cellular and molecular processes (Fig. 4 and Additional file 6: Table S3). The analysis of biological processes mediated by ZoAP2/ERFs depicted that a predominant of ZoAP2/ERF proteins were involved in positive regulation of nucleic acid-templated transcription, RNA biosynthetic process, and DAN-templated transcription; and stress responses, such as response to water deprivation, salt stress, and freezing. The molecular processes of ZoAP2/ERF proteins clearly showed that 150 of the 163 ZoAP2/ERF proteins possessed organic cyclic compound binding and transcription regulatory activities. Further, cellular component analysis revealed the localization of ZoAP2/ERF proteins in the intracellular organelle, membrane-bounded organelle, intracellular membrane-bound organelle, organelle and nucleus.

### Chromosomal distribution, gene duplication, and synteny analyses of ZoAP2/ERF genes

**Fig. 5:**
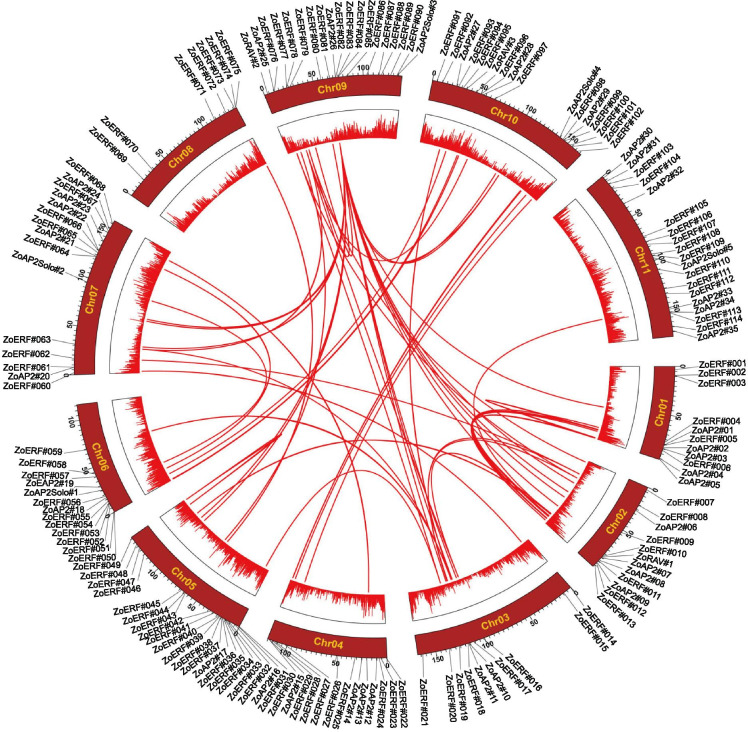
Schematic presentations of the inter-chromosomal relationships of ginger AP2/ERF genes. The red lines indicate duplicated AP2/ERF gene pairs in ginger. The chromosome number is indicated in the middle of each chromosome

Based on chromosome mapping analysis, a total of 157 AP2/ERF TFs were found unevenly distributed on 11 ginger chromosomes (Additional file 1: Fig. S1). Chromosomes 5, 9, and 11 contained the largest numbers of AP2/ERF TFs (21, 19, and 19, respectively), while chromosome 8 had the smallest number of AP2/ERF TFs (7 genes). Three RAV subfamily members were distributed on chromosomes 2, 9, and chromosome 10. Interestingly, some TFs with similar conserved sequences were located on the same chromosome. Similar patterns have been found in the *A. thaliana* [5], Chinese cabbage [30], and pepper genomes [34], which were thought to be caused by ancestral polyploidy events. Gene replication plays an important role in the occurrence of novel functions and gene family expansion. We analyzed the tandem duplication events of AP2/ERF genes in the ginger genome based on the criterion of two or more genes distributed within a 200-kb chromosomal region. Sixteen ZoAP2/ERF genes were clustered into eight tandem duplication regions among ginger linkage groups (LGs) 1, 3, 4, 7, 8, and 9 (Additional file 7: Table S4). Four tandemly duplicated gene pairs contained different motifs, with one pair (*ZoAP2#15-ZoERF#30*) located on LG4 and the other three (*ZoAP2#20-ZoERF#061*, *ZoAP2#21-ZoERF#064*, and *ZoAP2#22-ZoERF#066*) located on LG7, which indicates a hot spot of the ZoAP2/ERF gene distribution in LG7. In addition to tandem duplication events, 15 pairs of segmentally duplicated genes were found in the ginger genome. Analyses of homologous protein families are of great significance in establishing the kinship of species and predicting the function of new protein sequences. Many homologous genes were present on different chromosomes in ginger, indicating high conservation of the AP2/ERF gene family (Fig. 5). In brief, based on the above results, some ZoAP2/ERF genes might be produced by eight tandem duplication and 15 segmental duplication events, and these duplication events might be the main driving force of *ZoAP2/ERF* evolution. The approximate times of the segmental and tandem duplication events were dated to 2.7 million years ago (Ma) and 4.66 Ma, respectively.

### Evolutionary analysis of ZoAP2/ERF genes

**Fig. 6:**
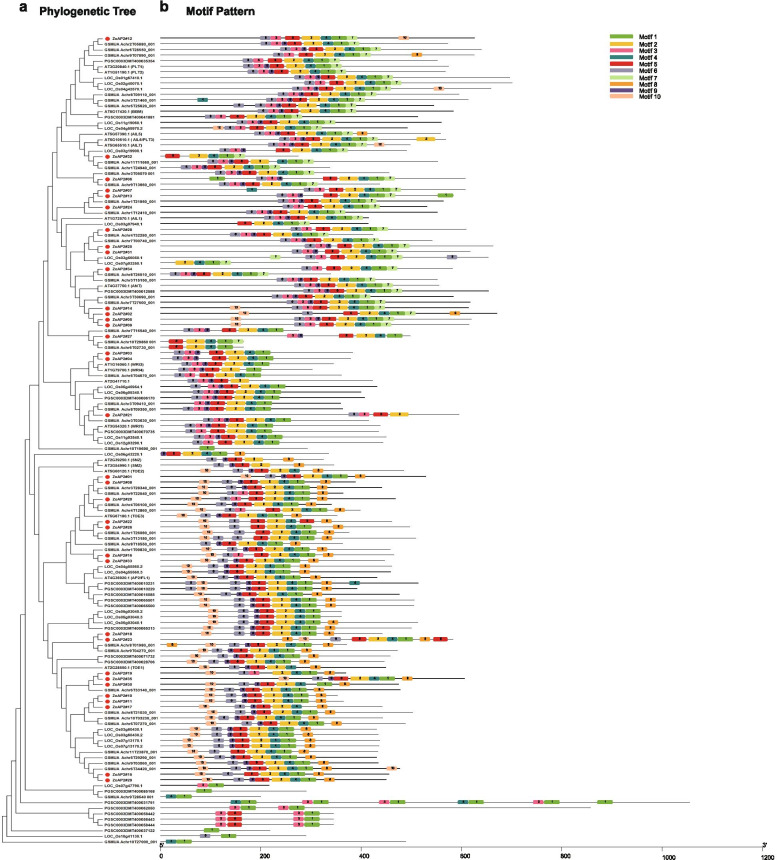
Phylogenetic relationships and motif compositions of AP2 proteins from five different plant species. Left panel: An unrooted phylogenetic tree constructed using MEGA X with the neighbor-joining method. The red solid circles represent AP2 genes from ginger. Right panel: Distribution of conserved motifs in AP2 proteins. The differently colored boxes represent different motifs and their position in each AP2 protein sequence

To deduce the evolutionary relationship of AP2/ERF genes, a phylogenetic tree of complete protein sequences from five species, including two dicotyledonous plants (*A. thaliana* and *S. tuberosum*) and three monocotyledonous plants (*O. sativa*, *M. acuminata,* and *Z. officinale*), was constructed. The AP2/ERF family of ginger contained three subfamilies: AP2, ERF, and RAV. To explore the evolutionary relationship of each gene, a phylogenetic tree was constructed among ginger and other plant members within each subfamily. Simultaneously, the motifs of the corresponding proteins were determined. As indicated in Fig. 4a, most members of the ginger AP2 subfamily were clustered with *M. acuminata* (33 members), followed by *A. thaliana* (two members). A total of 10 conserved motifs were detected in the protein sequences of the AP2 subfamily members in all the five plants (Fig. 6 and Additional file 8: Fig. S4). Almost all members contained Motif-1, Motif-2, Motif-3, Motif-4, Motif-5, and Motif-6. In addition, most AP2 members in the same clade usually shared common motif compositions, indicating potential functional similarities among AP2 subfamily proteins.

**Fig. 7:**
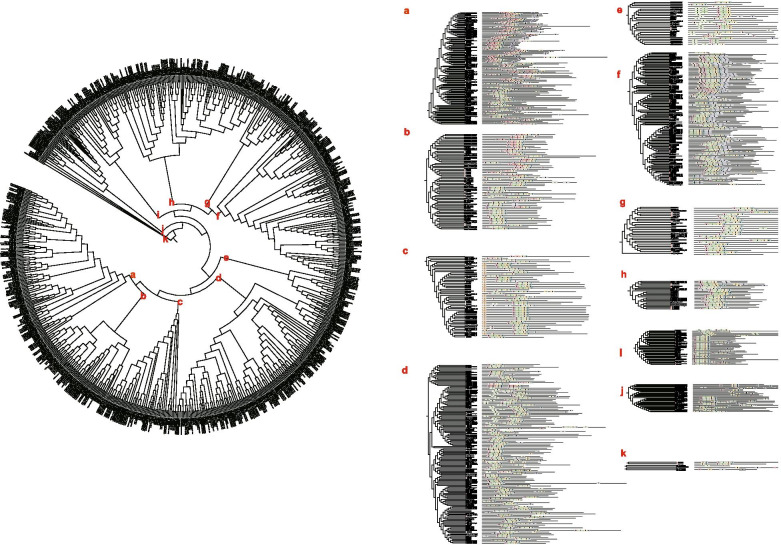
Phylogenetic relationships and motif compositions of ERF proteins from five different plant species. Left panel: An unrooted phylogenetic tree constructed using MEGA X with the neighbor-joining method. Right panel: Distribution of conserved motifs in ERF proteins. The differently colored boxes represent different motifs and their positions in each ERF protein sequence

The ERF subfamily of ginger contained 120 members, which were divided into 11 groups (Fig. 5). A total of 10 distinct motifs were identified in the ERF subfamily of all the five plants. All the members, excluding partial of Group-f, Group-m and Group-n, contained Motif-1, Motif-2, and Motif-3, and the genes that clustered together contained similar motifs. Motif-9 was unique in Group-a, and Group-e; Group-f contained Motif-5 specifically; Group-g and Group-i specifically Motif-4; Group-m specifically Motif-8; and Group-n specifically Motif-7 (Fig. 7 and Additional file 9: Fig. S5). The same method was used to analyze the clustering relationship between ZoRAVs and the RAV proteins of other plants. Moreover, the members of the same clade of the phylogenetic tree had almost the same motif compositions.

**Fig. 8:**
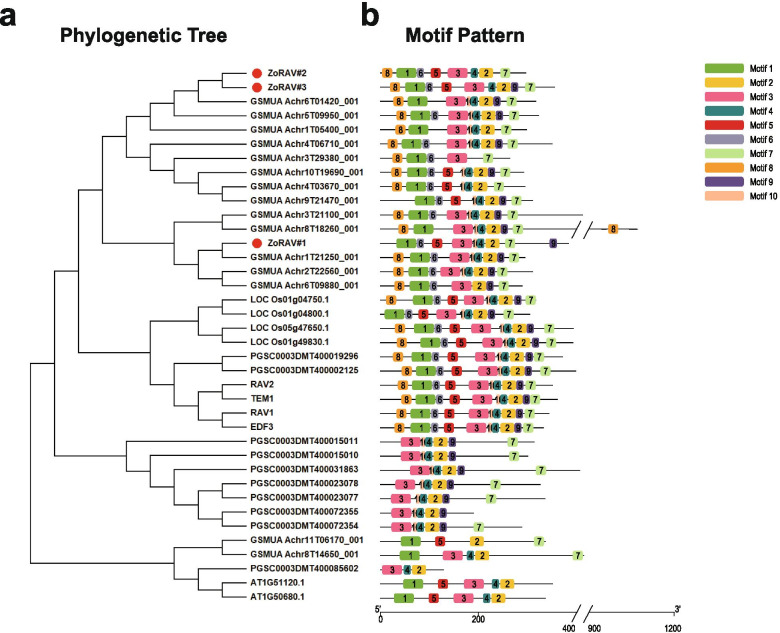
Phylogenetic relationships and motif compositions of RAV proteins from five different plant species.Left panel: An unrooted phylogenetic tree constructed using MEGA X with the neighbor-joining method.The red solid circles represent RAV genes from ginger. Right panel: Distribution of conserved motifs in RAV proteins. The differently colored boxes represent different motifs and their position in each RAV protein sequence

As illustrated in Fig. 8a, three ZoRAV genes were closely related to *RAV* genes in *M.acuminata*. The protein sequences of the RAV genes also showed 10 distinct conserved motifs, and most of the members contained Motif-2, Motif-3, and Motif-4 (Fig. 8 and Additional file 10: Fig. S6).

**Fig. 9:**
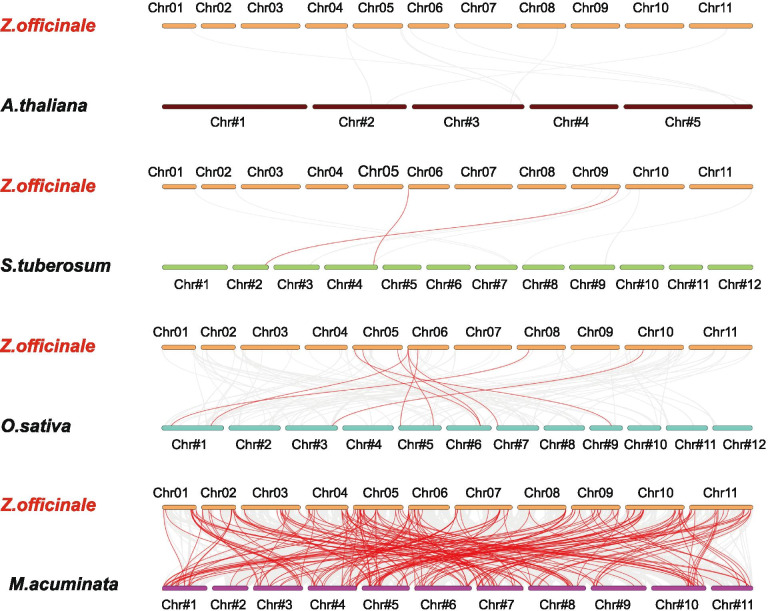
Synteny analysis of ERF genes between ginger and four representative plant species. The gray lines in the background indicate the collinear blocks between ginger and other plant genomes, while the red lines highlight the syntenic ERF gene pairs

To investigate the phylogeny of ginger AP2/ERF family, we constructed four comparative syntenic maps of ginger associated with other four representative plant species, including two dicots (Arabidopsis and potato) and two monocots (banana and rice). A total of 113 ZoAP2/ERF genes showed syntenic relationships with those in banana, followed by rice (8), potato (2), and Arabidopsis (0) (Fig. 9 and Additional file 11: Table S5). The numbers of orthologous pairs between ginger and the other four plant species (bananas, rice, potato and Arabidopsis) were 201, 9, 2, and 0, respectively. Some ZoAP2/ERF genes were found to be associated with at least three syntenic gene pairs (particularly between ginger and banana AP2/ERF genes), such as *ZoERF#027* and *ZoERF#096*, inferring that these genes may have played an vital role during the evolution of the AP2/ERF gene family. The AP2 subfamily genes in ginger showed homology to the reference plants, and the most syntenic conservation was observed in *M.acuminata* (201 orthologous gene pairs distributed on all LGs), *O.sativa* (9 orthologous gene pairs distributed on LG5, LG6, LG8, and LG10), and *S. tuberosum* (2 orthologous gene pairs distributed on LG6 and LG9) (Fig. 9 and Additional file 11: Table S5). The AP2/ERF genes were found in Arabidopsis; however, we did not locate any syntenic gene pairs between ginger and Arabidopsis. In the syntenic analysis of AP2/ERF genes between ginger and *M. acuminata*, 49 AP2/ERFs were found to be associated with two syntenic gene pairs, seven ginger AP2/ERFs were found to be associated with 3, 4 syntenic gene pairs and *ZoERF#027* was found to be associated with five syntenic gene pairs, indicating that these genes might play a vital role in AP2/ERF subfamily evolution.

Significantly, some highly conserved syntenic blocks between ginger and banana harbored more than 80 collinear genes. In contrast, those between ginger and rice were all located in syntenic blocks that possessed less than 50 orthologous gene pairs. Fewer gene pairs were found between ginger and Arabidopis, however, those did not include AP2/ERF family genes. We propose that the main reason lies in the recent whole genome duplication and tandem duplication events in the ginger genome.

To further investigate the evolutionary constraints acting on the AP2/ERF gene family, the Ka/Ks ratios of the AP2/ERF gene pairs were analyzed (Additional file 12: Table S6). All segmentally and tandemly duplicated ZoAP2/ERF gene pairs, as well as the majority of orthologous AP2/ERF gene pairs had Ka/Ks < 1, suggesting that the ginger AP2/ERF gene family might have experienced strong purifying selective pressure during evolution.

The syntenic analysis provided reliable evidence to sustain and validate the previous phylogenetic grouping and motif distribution. In summary, these data indicate that the ginger AP2/ERF gene family is highly conserved, and the ginger AP2/ERF genes are closer to the *M. acuminata* genes than to the *O. sativa* genes. The AP2/ERF genes might have evolved from a common ancestor of different plants.

### Expression profiling of ginger AP2/ERF genes in different tissues

**Fig. 10:**
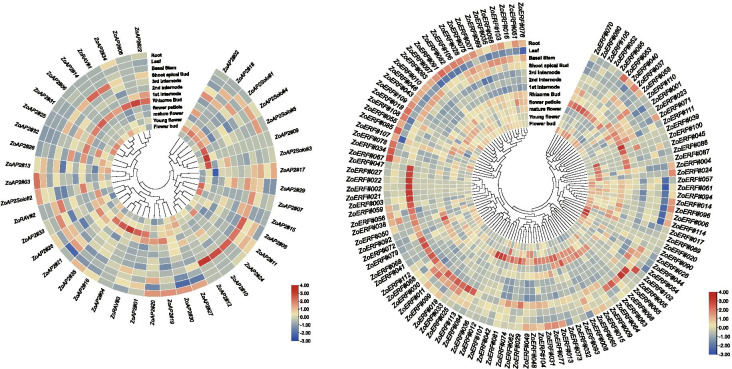
Expression profiles of the ginger AP2/ERF genes. Hierachical clustering of expression profiles of ginger AP2/ERF genes in 12 samples including different tissues and developmental stages

**Fig. 11:**
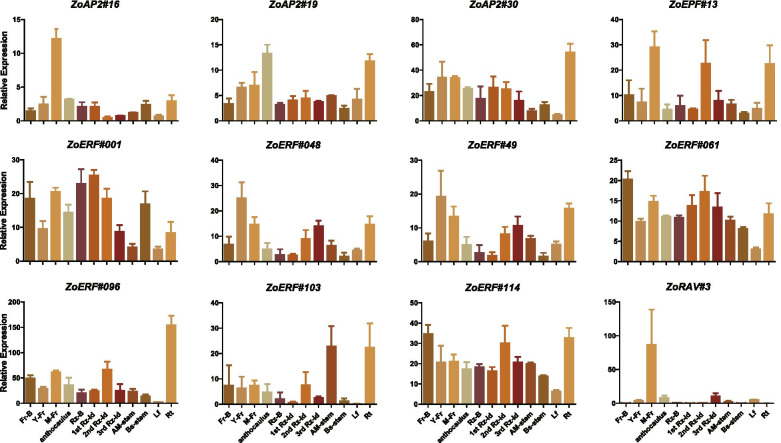
Expression analysis of 12 AP2/ERF genes in 12 samples by qRT-PCR. Data were normalized to *TUB-2* gene, and vertical bars indicate standard deviation

To investigate the potential functions of the ZoAP2/ERF genes in different developmental stages of ginger organs/tissues, we used RNA-seq data to detect their expression patterns (Fig. 10).The reliability of the transcriptome data was further validated by qRT-PCR experiments, which were carried out on eight representative samples for 12 selected ZoAP2/ERF genes (Fig. 11). Among the 35 ZoAP2 subfamily genes, *ZoAP2#23* was not expressed in any detected samples, indicating that it may be a pseudogene or has special temporal or spatial expression patterns not examined in our data. Seventeen ZoAP2 genes were expressed in all the 12 samples tested (FPKM > 0) and four ZoAP2 genes showed constitutive expression (FPKM > 1 in all samples). Some genes exhibited preferential expression across the detected tissues. Three genes in roots (*ZoAP2#17*, *ZoAP2#27*, and *ZoAP2#30*), two genes in mature florescences (*ZoAP2#16* and *ZoAP2#33*), one gene in anthocauli (*ZoAP2#19*), two genes in leaves (*ZoAP2#21*/33), and one genes in the meristem of stems (*ZoAP2#08*) showed the highest transcript expression levels. The expression of some genes exhibited significant trends in different developmental stages. For example, the expression levels of *ZoAP2#16/19/30/33*/*35* were gradually increased, whereas that of *ZoAP2#27* was gradually reduced along with the flower development (Fig. 10).

Among the 120 ZoERF subfamily genes, 69 were expressed in all the12 samples tested (FPKM > 0) and 22 showed constitutive expression (FPKM > 1 in all samples). Some genes displayed preferential expression across the detected tissues. Three genes in young flower buds (*ZoERF#004/048/049*), severn genes in mature flowers (*ZoERF#001/008/013/015/029/112/113*), three genes in top stems (*ZoERF#002/003/056*), 15 genes in roots (*ZoERF#007/014/028/051/061/078/082/091/096/097/103/106/107/108/.*

*113*), one gene in the second internode of rhizomes, eight genes in basal stems (*ZoERF#010*/*011*/ *019*/*044*/*076*/*082*/*089*) and four genes in the first bud of rhizomes (*ZoERF#006/035/052/061*) showed the highest transcript abundances. The expression of some genes exhibited significant trends in different developmental stages. For example, the expression levels of *ZoERF#15/30/31/60/085*/*106* were gradually increased, whereas those of *ZoERF#014/054/ 089/095* were gradually reduced during different developmental stages of the flower. The expression of *ZoERF#001/006/013/019/046/051/055/061/066/083/096* was reduced in flower emerging stage and then increased in flower elongation stage. The expression of *ZoERF#004*/*035*/*048* /*049*/*082/098* was increased in inflorescence emerging stage and then reduced in inflorescence elongation stage. Among the three ZoRAV subfamily genes, *ZoRAV#1* and *ZoRAV#2* showed no significant fluctuation with developmental stages, while *ZoRAV#3* was highly expressed in mature inflorescences (Fig. 10 and Additional file 13: Table S7)*.*

The transcriptional levels of all the163 *ZoAP2/ERF* genes in different whole-rhizome (rhizome bud, first inter-node, second inter-node, third inter-node) developmental stages were also investigated and the results showed that the expression of some ZoAP2/ERF genes was associated with the rhizome development in ginger. The transcripts of *ZoAP2#17/*19/27/30 were gradually reduced across different developmental stages of rhizome. The expression of *ZoERF#001/006/013/ 082/096/103/114* was increased between the rhizome bud and first rhizome inter-node stages, while it was reduced in the following developmental stages.

We examined the expression of 12 randomly selected genes using qRT-PCR in 12 different tissues. Overall, *ZoRAV#3* and *ZoAP2#16* were highly expressed in mature inflorescences than in other tissues, while *ZoERF#13* was more abundant in mature inflorescences, roots, and the second internode of rhizome. *ZoERF#096*, *ZoERF#103*, *ZoERF#114*, *ZoERF#119*, and *ZoAP2#30* were more abundant in roots than in other tissuses (Fig. 11). Theses results are consistent with the RNA-seq data.

### Expression patterns of ZoAP2/ERF genes in response to abiotic stress treatments

To investigate the potential functions of the ZoAP2/ERF genes under various abiotic stresses, we used RNA-seq data to detect their expression levels under cold, heat, drought, and salt treatments. Actually, 12 ZoAP2/ERF genes were not expressed in any of the four treatments. A total of 151 ZoAP2/ERF genes, including 31 ZoAP2s, 112 ZoERFs, three ZoRAVs and four ZoAP2Soloists, were induced by at least one stress treatment (Additional file 14: Fig. S6). In summary, the expression of 67 genes was induced by cold, that of 53 genes by heat, that of 50 genes by drought, and that of 61 genes by salt (Additional file 15: Fig. S7). Therefore, the greatest number of these genes was induced by cold and the lowest by drought. Additionally, there were 24, 18, 20, and 13 ZoAP2/ERF members down-regulated by cold, drought, heat, and salt treatments, respectively (Additional file 16: Fig. S8). Among these differentially expressed genes, 27 were induced, while two i.e., *ZoERF#050* and *ZoERF#065* were reduced under all four treatments, indicating that the ZoAP2/ERFs genes may be involved in a crosstalk between signal transduction pathways in response to different abiotic stresses, or that some functions of different genes are complementary.

We also examined the expression of 41 randomly selected genes using qRT-PCR under cold, heat, salt, and drought stress treatments. All the 41 selected genes were significantly induced by stress at one or more time point/s (Fig. 13), which was consistent with the RNA-seq data after 12 h of treatment. Overall, the gene response was slower in cold conditions than in drought conditions. Gene expression levels were gradually increased over time in low temperature conditions and reached a peak at 12 h or 24 h. In contrast, genes responded immediately to drought, with expression levels peaking at 1 h or 3 h after treatment (Fig. 13).

## Discussion

The AP2/ERF genes comprise a large family of TFs that are ubiquitous to all plant species and play vital roles in various physiological processes. They have been intensively studied in many plants, such as *A. thaliana* [5], popular [27]. However, no study has investigated the AP2/ERF genes in ginger. The genome-wide analysis of AP2/ERF family genes have been widely carried out in many species whose genomes have been sequenced [5, 26–35]. In this study, a search for AP2/ERF genes in the ginger genome resulted in the identification of 163 members, including 120 ERF subfamily members, 35 AP2 subfamily members, three RAV subfamily members, and five soloists, which were designated *ZoAP2#01* -*ZoAP2#35*, *ZoERF#001*-*ZoERF#125*, *ZoRAV#1*-*ZoRAV#3*, and *ZoAP2Solo#1-ZoAP2Solo#5* on the basis of their chromosomal location, respectively. Similar numbers of AP2/ERF genes in other plants were found. For example, rice has 157 AP2/ERF genes, including 139 ERF members; Arabidopsis has 145 AP2/ERF members, including 121 ERF genes. Moreover, the genome size was different among *O. sativa* ssp. *japonica* (466 Mb), Arabidopsis (125 Mb), and ginger (1.7Gb), indicating that the number of AP2/ERF family members is relatively stable and that there is no absolute correlation with genome size.

The gene structure of ZoAP2/ERF genes was assessed in this study. As shown in Fig. 2b, 63.03% (75/119) of ZoERF genes had no introns, while the number of introns in the AP2 subfamily genes ranged from 1 to 8. The gene structure of *AP2/ERF* genes in ginger was similar to that in tartary buckwheat [35]. The difference between the structure of the AP2 subfamily and ERF subfamily genes supports a vast differentiation in the genome evolution.

The domains and motifs of TFs are often related to DNA binding,protein interaction and transcriptional activities [37]. Motif analysis showed that the AP2 domain of the AP2/ERF genes in ginger contained Motif-1, Motif-2, Motif-4, and Motif-6 (Fig. 2c). Motif-3, Motif-7 and Motif-8 were specifically detected in different groups of the ERF subfamily, suggesting that they play an important role in this subfamily. The conserved structural domains of the ginger AP2/ERF proteins were assessed. Multiple sequence alignments revealed that 16 AP2/ERF proteins (ZoAP2#27/32 and ZoERF#033/068/075/080/082/088/089/ 079/093/108/113/104/117/120) had sequence variation in their AP2 domains. Most characterized AP2/ERF proteins exhibited binding preference to their cognate *cis*-acting GCC-box or DRE motifs with the assistance of the AP2 domain. In previous studies, variations in the YRGVR motif of the AP2 domain might influence normal interactions of AP2/ERF genes with downstream target genes, and therefore these 16 AP2/ERF proteins might be worthy to further characterize their functions and binding specificities [33, 38].These results propose that although some motifs/domains of the AP2/ERF family genes were highly conserved, the newly evolved motifs/domains might harbor new functions in specific plants, and the functions of these newly evolved motifs/domains require further verification.

Chromosomal fragments and duplications of individual genes or entire genomes have long been considered a major source of evolution, including novel functions and expression patterns [39]. In ginger, we found the ZoAP2/ERF family expanded distinctively, which was mainly contributed by segmental duplications (Figs. 5 and 9). Most duplicated ZoAP2/ERF genes were expressed in different tissues/organs, indicating that these genes have specific or redundant cellular functions during ginger development. Evidence for differences among duplicated genes can be deduced from the expression patterns of the *ZoERF#074* and *ZoERF#075* genes. *ZoERF#074* was highly expressed in mature inflorescences and basal stems, but *ZoERF#075* was not expressed in any organ/tissues. The composition and position of their motifs (Motif-4, Motif-2, Motif-1, Motif-6) were identical (Fig. 8), and thus we conjecture that the reason for their different expression pattern might be due to the mutation of genes involved in the process of duplication, which leads to a loss of function of *ZoERF#077*.

Additionally, functional differences may bring about differences in gene pair expression patterns. For instance, the mRNA abundance of *ZoERF#061* peaked in the bud of inflorescences, but *ZoAP2#20* was highly expressed in roots (Additional file 10: Fig. S6). *ZoAP2#20* (Motif-4, Motif-9, Motif-5, Motif-3, Motif-1, Motif-6) contained Motif-3, Motif-5, and Motif-9, but *ZoERF#062* (Motif-4, Motif-2, Motif-1, Motif-6) did not (Fig. 8). Therefore, we speculate that the alteration of the motifs of the two genes during duplication may be one of the reasons to the functional differences.

**Fig. 12:**
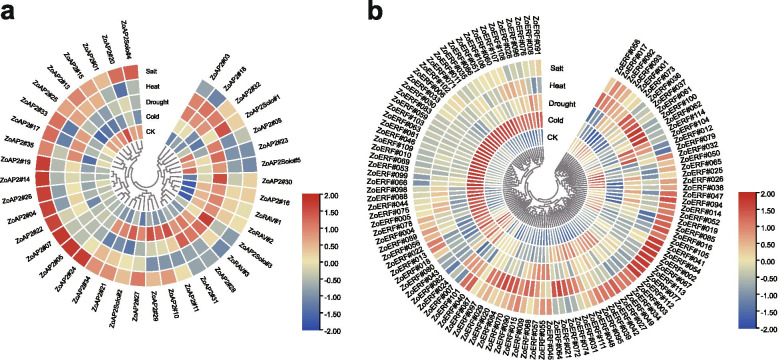
Expression profiles of ZoAP2/ERF genes under abiotic stress treatments. **a** AP2, RAV and Soloist subfamily. **b** ERF subfamily

Gene function can be preliminarily predicted by analysis of the gene expression profiles [37]. Tissue-specific expression patterns indicated that most of the AP2 subfamily genes (17/35, 49.6%), ERF subfamily genes (75/119, 63.0%) (Fig. 8), and RAV subfamily genes (2/3, 66.7%) (Fig. 12) were expressed in all tested tissues. However, 22 ZoAP2/ERFs were expressed at higher abundance in roots, and similar results were found in other plant species [27, 29]. Ginger is usually not easy to blossom, sometimes once in 10 years in cultivation. However, the flowering mechanism of ginger is still unclear. In model plants such as *A. thaliana*, transformation of vegetative to reproductive growth has been intensive studied, and the AP2/ERF family is involved in controlling flowering. In our study, 49 ZoAP2/ERF genes were abundantly expressed in inflorescence development. According to the phylogenetic tree, we found AP2 genes (*ZoAP2#01*, *ZoAP2#16*, and *ZoAP2#33*) that were highly expressed in mature inflorescences were clustered together (Fig. 1). Exploration of the evolutionary relationship between these AP2 genes and AP2 genes in other plants revealed a similar evolutionary relationship and identical motif compositions between *ZoAP2#16/33* and *At4G36920.1* (*FL1*) (Fig. 4). Moreover, *FL1* was identified as a gene participating in the specification of floral organ identity in *A. thaliana*, which provides a direction for us to further characterize the function of *ZoAP2#16/33* [40]. In ginger, *ZoAP2#16/33* had relative higher expression in inflorescences compared to leaves and other vegetative organs, indicating that *ZoAP2#16/33* may be also associated with the regulation of flower development. Indeed, it is essential to further validate whether *ZoAP2#16/33* can also promote inflorescence initiation and growth in ginger. In Arabidopsis, ANT (At4G37750) and AIL6(At5g10510) are key regulons in floral organ positioning, identity, and growth [41–43]. However, the homologs of Arabidopsis *ANT* and *AIL6* in ginger (*ZoAP2#02/05/09/14* and *ZoAP2#32*) were not expressed in flower initiation and growth phases, suggesting the functional divergence of AP2 genes during evolution. Motif composition analysis indicates that ZoAP2#02/05/09/14 gained an extra motif element (Motif-10) in the N -terminus compared with its homolog ANT, while ZoAP2#32 has lost Motif-6/3/9 compared with its homolog AIL6 in Arabidopsis (Fig. 4). It is supposed that motif gain and loss may result in function divergence.Therefore, we need to further screen the AP2 /ERF genes possessing functions similar to *ANT* or *ANT-like* genes that play an important role in regulating the initiation and development of flowers in ginger.

*ZoERF#013* exhibited higher expression in all floral developmental stages and the highest expression in mature inflorescences.Considering its otholog in Arabidopsis, *ERF12*, is involved in regulating the floral meristem identity [44], *ZoERF#006* may share similar functions in ginger.

Rhizome is the economic organ of ginger, and the process of rhizome enlargement is the focus of attention in cultivation. Zhou et al. (2016) reported that *AtERF11* (*At1g28370*) promotes internode elongation by promoting both GA biosynthesis and signaling pathways [45]. Based on the phylogenetic analysis, *ZoERF#064* was an ortholog of *AtERF11. ZoERF#006* was also highly expressed in the internodes of rhizome, which is probably related to the elongation of rhizome. ERF139 is a key factor within a negative regulatory cascade that controls vessel expansion in poplar [46]. In ginger, *ZoERF#70* is an ortholog of *ERF139*; according to the expression pattern, it showed the highest expression in leaf buds, and its expression gradually reduced from the tender to old internodes during the rhizome growth and development. Therefore, we need to further verify whether *ZoERF#006* possesses a function similar to *ERF139* and plays a vital role in regulating the growth and development of rhizome.

**Fig. 13:**
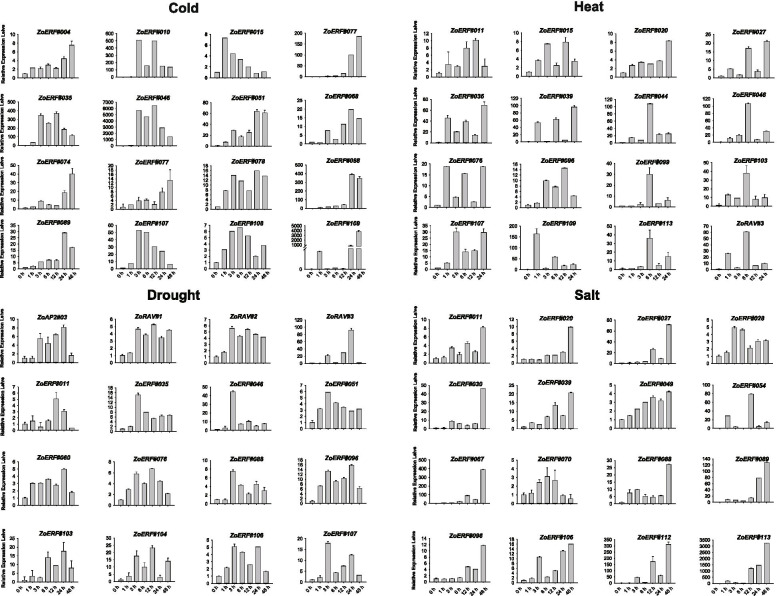
Expression analysis of AP2/ERF genes under abiotic stresses by qRT-PCR. Data were normalized to *TUB-2* gene, and vertical bars indicate standard deviation

The AP2/ERF family genes are known to be involved in diverse processes of environmental stress responses, such as cold, heat, drought, and salinity [5, 47, 48]. *CBF1*, *CBF2*, and *CBF3*, belonging to the A1 group of the DREB subfamily, are cold-inducible at low temperatures [49–51]. Arabidopsis showed stronger cold tolerance than wild-type plants by overexpressing the *CBF1*, *CBF2*, and *CBF3* genes of *Brassica. napus* [52]. In ginger, *ZoERF#010*, *ZoERF#046*, *ZoERF#077*, *ZoERF#078*, and *ZoERF#083* were classified to the DREB-A1 group. *ZoERF#010*, *ZoERF#083*, and *ZoERF#046* were dramatically induced by cold, whereas *ZoERF#077* and *ZoERF#078* did not significantly respond to cold treatment. However, the expression patterns of *ZoERF#010*, *ZoERF#083*, and *ZoERF#046* were different in response to cold treatment. *ZoERF#046* quickly responded to low temperatures at 1 h, peaked at 12 h, and then reduced gradually within 24-48 h (Fig. 13). *ZoERF#010* and *ZoERF#083* did not significantly accumulate until 3 h after treatment. Additionally, *ZoERF#046* also could be induced by drought, heat, and salinity stress treatments. Indeed, the *cis*-elements of *ZoERF#046* contained TC-rich repeats that are responsible for defense and stress responsiveness. The other 59 ZoAP2/ERFs were also induced by cold treatment, but the expression levels of these genes were lower, indicating that *ZoERF#010*, *ZoERF#083*, and *ZoERF#046* play an essential role in cold response.

*ZoERF#18*, *ZoERF#20*, *ZoERF#52*, *ZoERF#95*, and *ZoERF#102* belonged to the A2 group of the DREB subfamily. Most genes in this group were not induced by heat treatment, except for *ZoERF#20* and *ZoERF#95*. These results are different from those in other plants [52]. Interestingly, *ZoERF#95* and *ZoERF#102* were induced by salinity, whereas *ZoERF#18* was responsive to cold stress treatment. In this study, we found 27 ZoAP2/ERFs were responsive to multiple abiotic stresses, which suggests that these ZoAP2/ERFs may act as the key nodes of signal pathways in response to environmental fluctuation.

Overall, the above findings provide insight into the potential functional roles of ginger AP2/ERF genes. The comprehensive analyses are beneficial to screening candidate AP2/ERF genes for further functional characterization and genetic improvement in the agronomic characters of ginger.

## Conclusions

A comprehensive analysis of the AP2/ERF family genes in ginger was carried out in this study. A total of 163 full-length AP2/ERF genes were identified and further classified into three subfamilies, with highly similar gene structure and motif compositions in the same subfamily or subgroup. Phylogenetic comparison and synteny analysis of AP2/ERF genes from various plants provided valuable clues about the evolutionary characteristics of AP2/ERFs in ginger. ZoAP2/ERF genes played vital roles in ginger growth and development as indicated by their expression patterns. The phylogeny and gene expression analyses in the development of ginger rhizome and abiotic stress treatments will shed light on the functional analysis of ZoAP2/ERF genes. In short, these results provide a valuable resource for better understanding the biological roles of individual AP2/ERF genes in ginger.

## Materials and methods

### Gene identification and classification

The largest number of AP2/ERF genes were found in ginger genome (data from our ginger genome research project) by two BLASTP methods. The candidate genes were searched by BLASTP using a score value of ≥100 and e-value of ≤ e-10. Then the HMM files from the Pfam protein family database (http://pfam.xfam.org/) corresponding to the AP2 domain (PF00847) and the B3 domain (PF02362) were downloaded. AP2/ERF genes were retrieved from the ginger genome database by HMMER v3.0. The default parameters were applied and the cutoff was set to 0.01. The Pfam and SMART databases were used to determine the existence of AP2 core sequences. Finally, 163 AP2/ERF gene models were identified in the ginger genome for further analyses. The basic information of the identified AP2/ERF proteins was obtained using the tools at the ExPasy website (http:// web.expasy.org/protparam/).

### Sequence analysis

The structural differences among *Z. officinale* AP2/ERF (ZoAP2/ERF) genes were investigated by studying the conserved motifs of the AP2/ERF proteins. The ZoAP2/ERF protein sequences were aligned with ClustalW with default parameters. The exon-intron structure of the ZoAP2/ERF genes was determined by Gene Structure Display Server (GSDS: http://gsds.cbi.pku.edu.cn/) and the conserved motifs of AP2/ERF proteins were evaluated by the MEME online program (http:/meme.nbcr.- net/meme/intro.html) [53].

### Chromosomal distribution and duplication of ZoAP2/ERF genes

The method of mapping ZoAP2/ERF genes to the chromosomes of ginger was according to Xing et al. [54]. The analysis of gene duplication events was conducted by using multiple collinear scanning toolkits (MCScanX). The syntenic analysis maps of the Dual Systeny Plotter software were constructed to determine the AP2/ERF syntenic relationship between ginger and banana, potato, rice, and Arabidopsis, respectively [55].

### Phylogenetic analysis and classification of the *ZoAP2/ERF* gene family

According to the number of the AP2 conserved domain and the existence of the B3 domain, ZoAP2/ERF genes were divided into different groups. The AP2/ERF protein sequences of *A. thaliana*, *S. tuberosum*, *O. sativa* and *M. acuminata* were downloaded from the UniProt database (https://www.uniprot.org/). Multiple sequence alignments of AP2/ERF protein sequences from ginger and Arabidopsis were generated using MUSCLE with default parameters. The obtained multiple sequence alignments were used to construct a ML phylogenetic tree using MEGA X program with partial deletion, 80% cutoff, the JTT + G + I + F amino acid substitution model, and 1000 bootstrap replicates. The obtained ML tree was uploaded to iTOL (https://itol.embl.de/) [34] to draw the dendrogram.

### Plant materials

The *Z. officinale* accessions ‘LAIWU No.2’ was used in this study. For the expression analyses of ZoAP2/ERF genes, six-month-old seedlings including ginger flowers, flower buds, anthocauli, stems, rhizome buds, 1st, 2nd and 3rd rhizome internodes, functional leaves (third leaves from the apical to basal stem), leaf buds, and roots were collected. To investigate the function of ZoAP2/ERF genes in response to different abiotic stresses, two-month-old seedlings with five leaves were used. For drought and salinity treatments, the plant was watered with 15% PEG6000 and 200 mM NaCl solution, respectively. The ginger plantlets were subjected to 40 °C and 4 °C for heat and cold stress treatments, respectively. The leaf samples were collected at 0, 1, 3, 6, 12, 24, and 48 h after cold, drought, and salt treatments, respectively. The leaves were collected at 0, 1, 3, 6, 12, and 24 h after heat treatment. The collected samples were quickly placed in liquid nitrogen and stored at − 80 °C for subsequent analyses.

### Expression analysis of ZoAP2/ERF genes by RNA-seq and qRT-PCR

Samples collected at 12 h after cold, heat, drought, and salt treatments were used for RNA-seq. Total RNA was isolated using the TRIzol kit (Invitrogen, USA) and purified using an mRNA purification kit (Promega, China) following the manufacturers’ protocols. Approximately 20 μg of total RNA from each sample was enriched using oligo (dT) magnetic beads and digested into short fragments, and then the first- and second-strand cDNA were synthesized at the BGI (Shenzhen, China). After purification, the fragments were ligated to the sequencing adaptors and sequenced on an Illumina Hiseq 2000 sequencing system. A rigorous algorithm was used to identify differentially expressed genes in the ginger defense response. The false discovery rate (FDR) was set at 5% to determine the *P* value threshold for multiple comparison tests and analyses by manipulating the FDR value. *P* < 0.001 and the absolute value of log2 ratio > 1 were used as thresholds to judge the significance of differences in the gene expression. Based on the *Z. officinale* (“Zhugen” cultivar) genome sequence, we screened the corresponding sequences of ZoAP2/ERF genes. Meanwhile, the qRT-PCR primers were designed using Primer Primier 5 software (http://frodo.wi.mit.edu/) (Additional file 17: Table S9). The spatial and temporal expression and abiotic stress response of selected ZoAP2/ERF genes were analyzed by qRT-PCR. *TUB2* gene is expressed in almost all tissues with little difference in expression levels and is often used as an internal reference gene. The *ZoTUB2* gene was used as an internal control, and each qRT-PCR experiment with SYBR Premix Ex Taq II (TaKaRa) was performed at least three times using a CFX96 Real Time System (Bio-Rad). The reaction was carried out as follows: 95 °C for 30 s, followed by 40 cycles of 95 °C for 10 s and 60 °C for 30 s. Each reaction was performed in three biological replicates.The experimental data were processed by the 2^-△△CT^ method [56].

### Statistical analysis

All the data were analyzed by analysis of variance using the Sigma Plot v10.0 (SYSTAT software, USA) statistics program, and the means were compared by the Fisher’s least significant difference (LSD) test at significance levels of 0.05 and 0.01, respectively.

## Supplementary Information

**Additional file 1: Figure S1.** Schematic representations for the chromosomal distribution of ginger AP2ERF genes.

**Additional file 2: Table S1.** List of the 163 ZoAP2ERF genes identified in this study.

**Additional file 3: Figure S2.** Alignment of multiple ZoAP2ERF and selected AP2 domain amino acid sequences.

**Additional file 4: Figure S3.** Analysis and distribution of conserved motifs in ginger AP2ERF proteins.

**Additional file 5: Table S2.** Summary of cis-elements is in the promoter regions of ZoAP2/ERF family genes in ginger.

**Additional file 6: Table S3.** GO annotation of ZoAP2/ERF.

**Additional file 7: Table S4.** List of the tandem repeat gene pairs of AP2ERF in ginger.

**Additional file 8: Figure S4.** Analysis and distribution of conserved motif in AP2 subgroup of ginger and other species.

**Additional file 9: Figure S5.** Analysis and distribution of conserved motif in ERF subgroup of ginger and other species.

**Additional file 10: Figure S6.** Analysis and distribution of conserved motif in RAV subgroup of ginger and other species.

**Additional file 11: Table S5.** The lists of ginger and other species AP2ERF synteny gene pairs.

**Additional file 12: Table S6.** Ka/Ks ratio.

**Additional file 13: Table S7.** Expression profile of ZoAP2/ERF family genes in different tissues.

**Additional file 14: Table S8.** Expression profile of ZoAP2/ERF family genes response to abiotic stress treatment.

**Additional file 15: Figure S7.** The Venn map of up regulated genes of ZOAP2/ERF family response to different abiotic stresses.

**Additional file 16: Figure S8.** The Venn map of down regulated genes of ZOAP2/ERF family response to different abiotic stresses.

**Additional file 17: Figure S9.** The primer sequences for qRT-PCR used in this study.

## Data Availability

The datasets used and/or analyzed during the current study are available from the corresponding author on reasonable request.
