# Supplementary figures and images for: Genome-wide investigation of the AP2/ERF gene family in ginger: evolution and expression profiling during development and abiotic stresses
Source: BMC Plant Biol. 2021 Nov 25;21:561. doi: 10.1186/s12870-021-03329-3 (PMC8620233; doi:10.1186/s12870-021-03329-3)

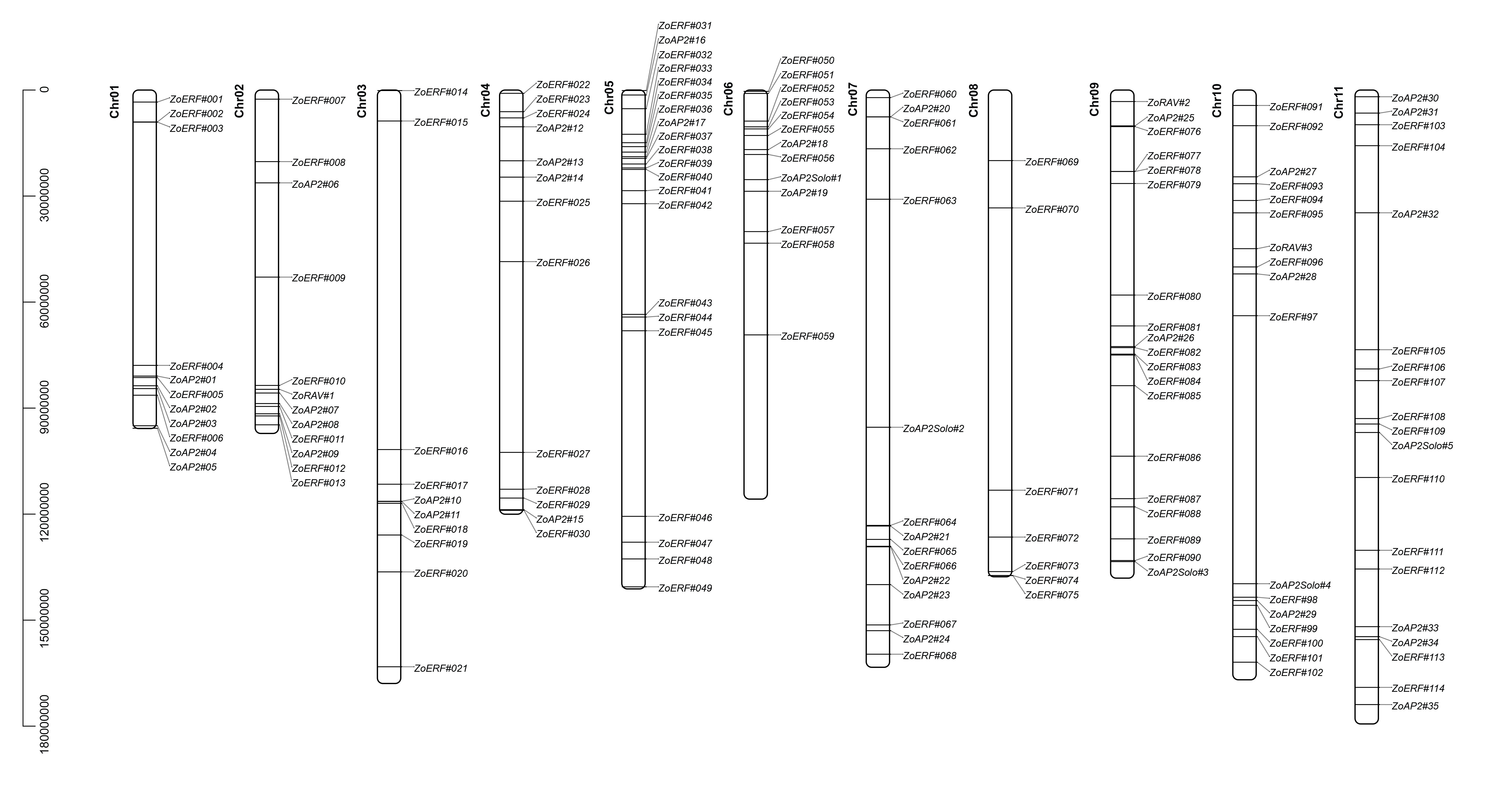

Supplement: Supplementary file 1 — Additional file 1: Figure S1. Schematic representations for the chromosomal distribution of ginger AP2ERF genes. [file 12870_2021_3329_MOESM1_ESM.jpg]

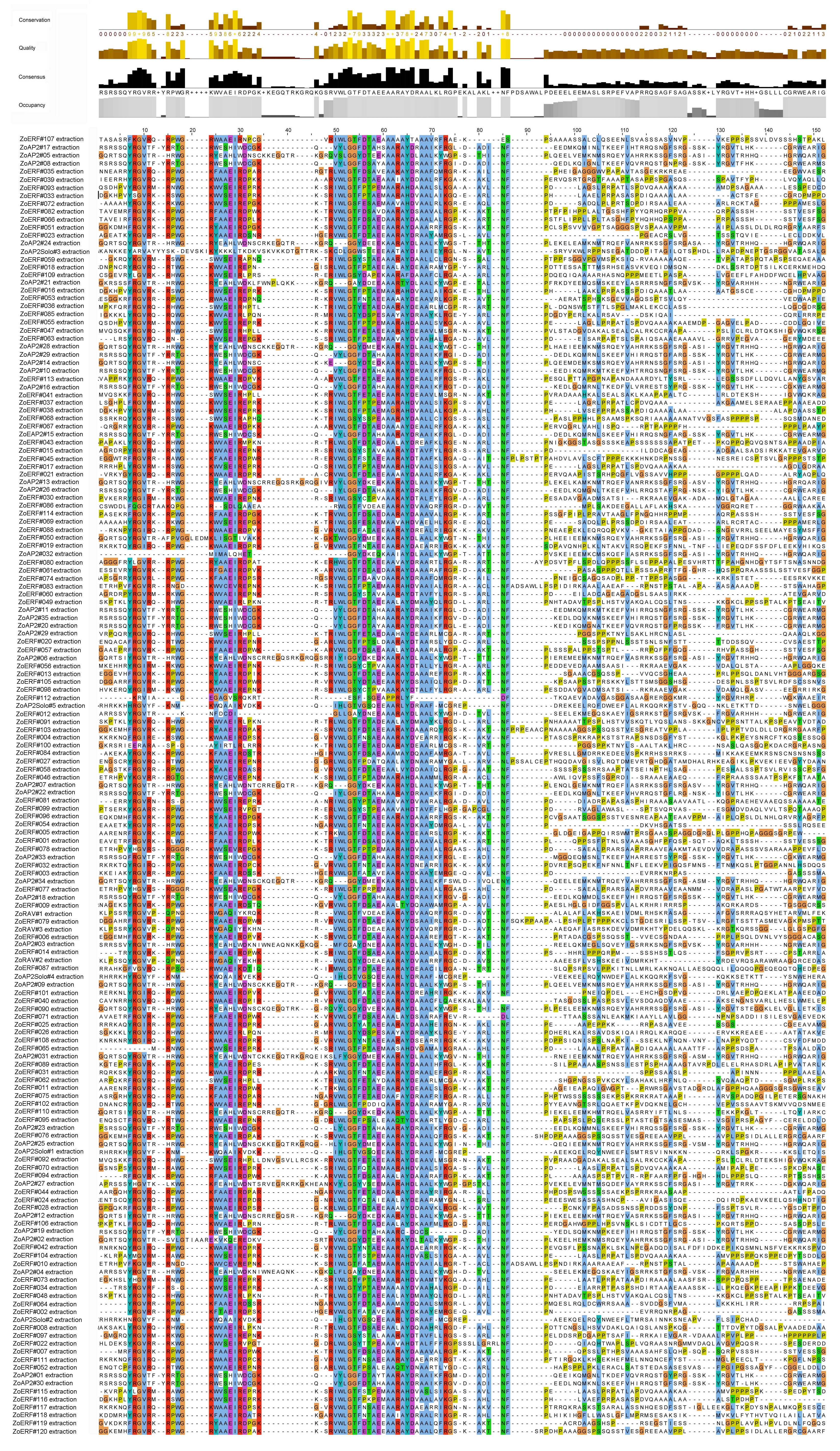

Supplement: Supplementary file 3 — Additional file 3: Figure S2. Alignment of multiple ZoAP2ERF and selected AP2 domain amino acid sequences. [file 12870_2021_3329_MOESM3_ESM.jpg]

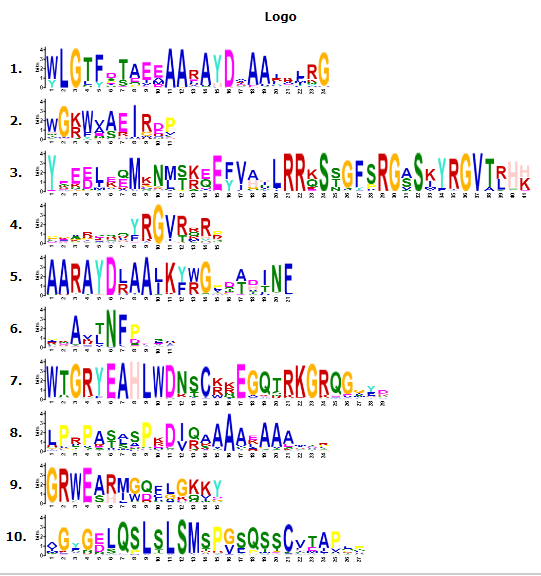

Supplement: Supplementary file 4 — Additional file 4: Figure S3. Analysis and distribution of conserved motifs in ginger AP2ERF proteins. [file 12870_2021_3329_MOESM4_ESM.jpg]

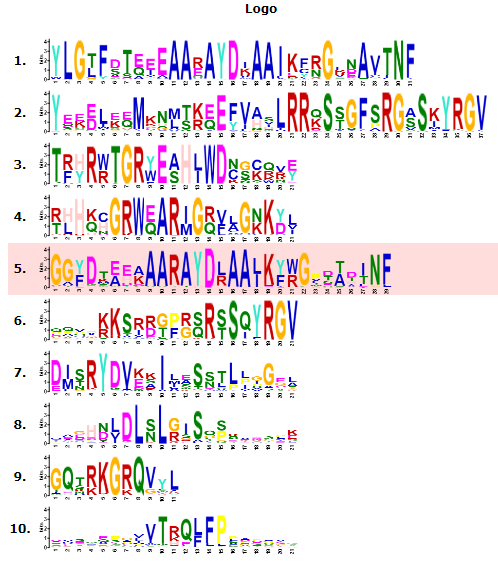

Supplement: Supplementary file 8 — Additional file 8: Figure S4. Analysis and distribution of conserved motif in AP2 subgroup of ginger and other species. [file 12870_2021_3329_MOESM8_ESM.png]

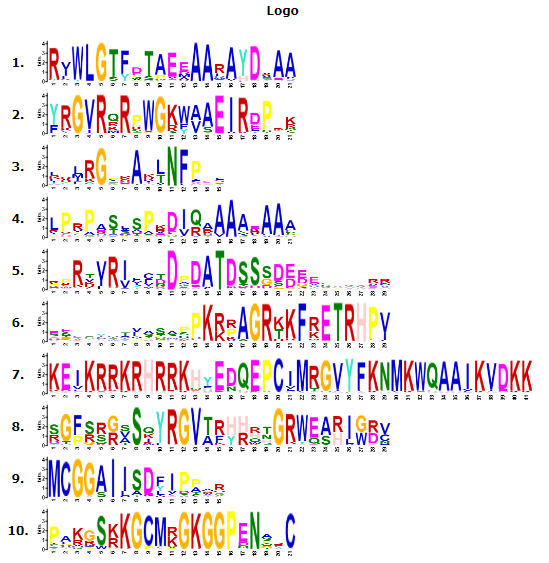

Supplement: Supplementary file 9 — Additional file 9: Figure S5. Analysis and distribution of conserved motif in ERF subgroup of ginger and other species. [file 12870_2021_3329_MOESM9_ESM.png]

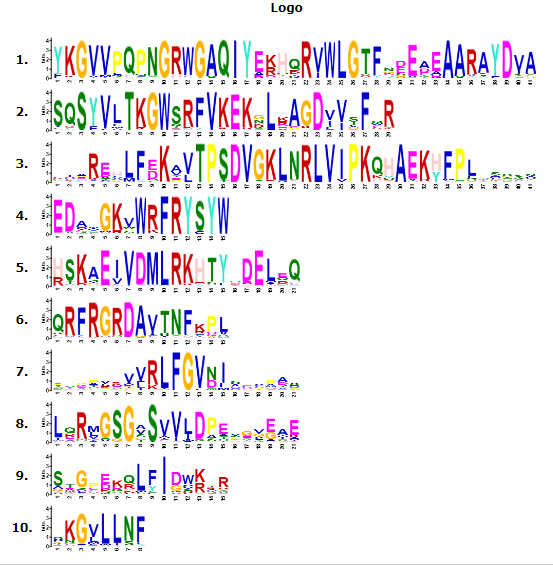

Supplement: Supplementary file 10 — Additional file 10: Figure S6. Analysis and distribution of conserved motif in RAV subgroup of ginger and other species. [file 12870_2021_3329_MOESM10_ESM.png]

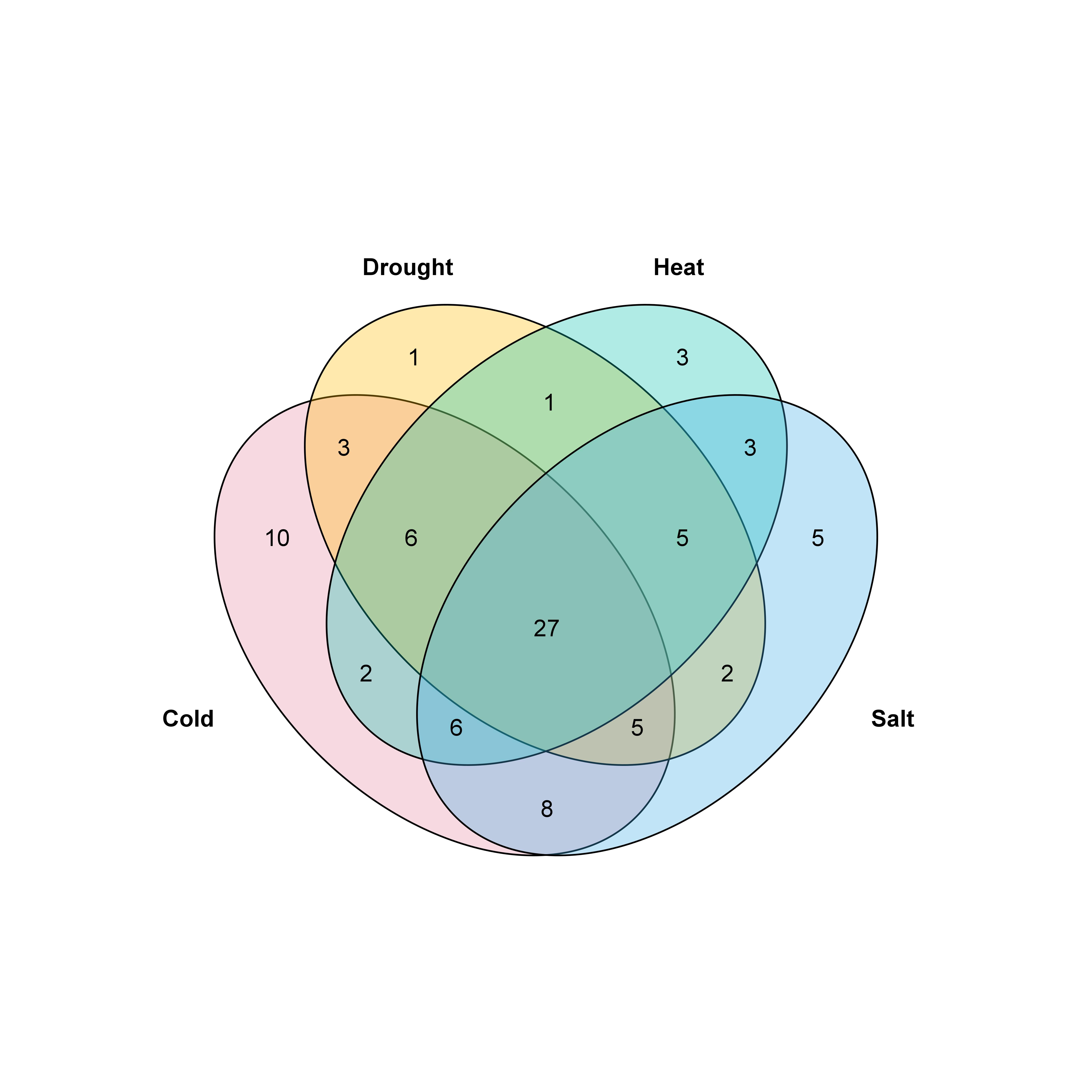

Supplement: Supplementary file 15 — Additional file 15: Figure S7. The Venn map of up regulated genes of ZOAP2/ERF family response to different abiotic stresses. [file 12870_2021_3329_MOESM15_ESM.jpg]

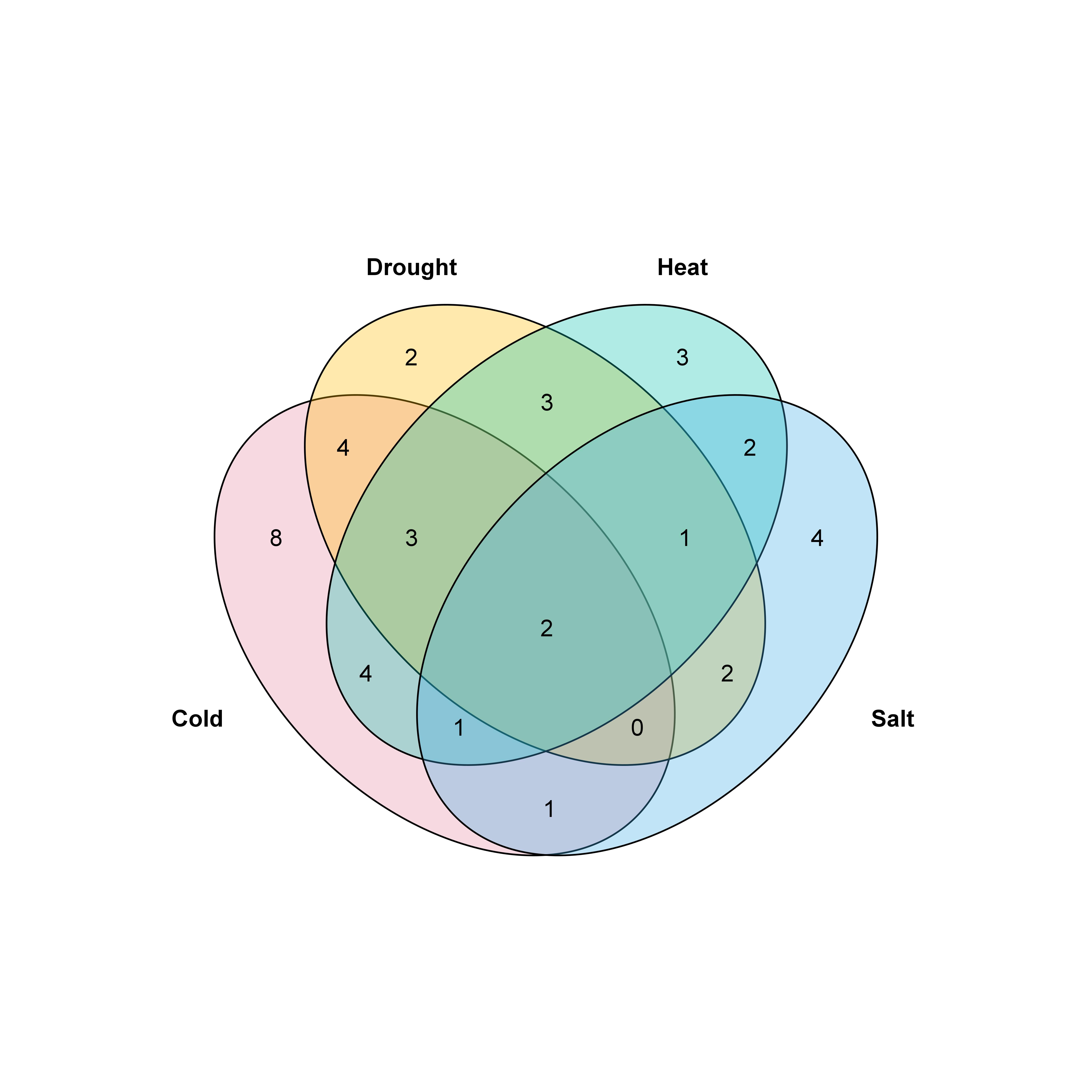

Supplement: Supplementary file 16 — Additional file 16: Figure S8. The Venn map of down regulated genes of ZOAP2/ERF family response to different abiotic stresses. [file 12870_2021_3329_MOESM16_ESM.jpg]
